# Supplementary material for: Sustained correction of hippocampal neurogenic and cognitive deficits after a brief treatment by Nutlin-3 in a mouse model of fragile X syndrome
Source: BMC Med. 2022 May 13;20:163. doi: 10.1186/s12916-022-02370-9 (PMC9103116; doi:10.1186/s12916-022-02370-9)
Supplement: Supplementary file 2 — Additional file 2: Code S2. R codes for differentially expressed gene analysis. [file 12916_2022_2370_MOESM2_ESM.pdf]

STAR alignment parameters

```
##### Final user re-defined parameters-----------------:
runThreadN                        4
readFilesCommand                  zcat
outSAMtype                        BAM   SortedByCoordinate
outSAMattributes                  NH   HI   AS   NM   MD
outSAMunmapped                    Within
outFilterType                     BySJout
quantMode                         TranscriptomeSAM   GeneCounts
twopassMode                       Basic
```
