# Supplementary material for: Sustained correction of hippocampal neurogenic and cognitive deficits after a brief treatment by Nutlin-3 in a mouse model of fragile X syndrome
Source: BMC Med. 2022 May 13;20:163. doi: 10.1186/s12916-022-02370-9 (PMC9103116; doi:10.1186/s12916-022-02370-9)

Differential expression analysis (DESeq2)

Input dataset

```
library("DESeq2")
library( "biomaRt" ) #example code for mouse gene id mapping

rm(list=ls())
setwd("~/Desktop/Waisman/Xinyu/counts/")

## Read dataset
counts= as.matrix(read.csv("./read_count.csv",row.names = 1))

condition = c("Vehicle","Vehicle","Vehicle",
              "Vehicle","Vehicle","Vehicle",
              "Nutlin_3","Nutlin_3","Nutlin_3",
              "Nutlin_3","Nutlin_3","Nutlin_3")
type = c("wildtype","wildtype","wildtype",
         "knock_out","knock_out","knock_out",
         "wildtype","wildtype","wildtype",
         "knock_out","knock_out","knock_out")
condition = factor(condition,levels = c("Vehicle","Nutlin_3"))
type = factor(type,levels = c("wildtype","knock_out"))
coldata = data.frame(type = type, condition = condition, row.names = colnames(counts))
```

```
#ensembl.genes=rownames(counts)
#ensembl = useMart( "ensembl", dataset = "mmusculus_gene_ensembl" )
#genemap <- getBM( attributes = c("ensembl_gene_id", "mgi_symbol","external_gene_name"), filters = "ensembl_gene_
id",values =ensembl.genes, mart = ensembl )
#counts=counts[ensembl.genes%in%genemap$ensembl_gene_id,]
#counts=counts[genemap$ensembl_gene_id,]
#rownames(counts)=genemap$external_gene_name
#counts["Fmr1",]
```

```
counts=counts[rowSums(counts) > 20,]
dds <- DESeqDataSetFromMatrix(countData = counts,
                              colData = coldata,
                              design = ~ condition + type + condition:type)
```

Detect DE genes between KO_Vehicle and KO_Nutlin3

```
subset1_idx=(as.character(type)=="knock_out")
dds_1 <- DESeqDataSetFromMatrix(countData = counts[,subset1_idx],
                              colData = coldata[subset1_idx,],
                              design = ~ condition)
dds_1 <- DESeq(dds_1)
res_1 <- results(dds_1)
summary(res_1) #up: 1.5%, down: 0.036%
```

```
##
## out of 22583 with nonzero total read count
## adjusted p-value < 0.1
## LFC > 0 (up)       : 337, 1.5%
## LFC < 0 (down)     : 8, 0.035%
## outliers [1]       : 17, 0.075%
## low counts [2]     : 4379, 19%
## (mean count < 6)
## [1] see 'cooksCutoff' argument of ?results
## [2] see 'independentFiltering' argument of ?results
```

```
# Result is stored in "resOrdered1"
resOrdered1=res_1[order(res_1$padj),]
#write.csv(as.data.frame(resOrdered1), file="Veh_vs_Nut(KO).csv") # this line is to write result into .csv file
#resLFC_1 <- lfcShrink(dds_1, coef="condition_Nutlin_3_vs_Vehicle", type="apeglm")

# check the first 5 lines of result
head(resOrdered1)
```

```
## log2 fold change (MLE): condition Nutlin 3 vs Vehicle
## Wald test p-value: condition Nutlin 3 vs Vehicle
## DataFrame with 6 rows and 6 columns
##                     baseMean log2FoldChange     lfcSE      stat      pvalue
##                    <numeric>      <numeric> <numeric> <numeric>   <numeric>
## ENSMUSG00000029843   355.277        2.74431  0.202111  13.57825 5.39018e-42
## ENSMUSG00000095562   108.846       -6.00510  0.506700 -11.85140 2.11620e-32
## ENSMUSG00000039004   281.669        1.92368  0.165399  11.63054 2.88265e-31
## ENSMUSG00000004105   125.551        2.23619  0.234225   9.54719 1.33262e-21
## ENSMUSG00000022150   260.880        1.50927  0.158172   9.54197 1.40150e-21
## ENSMUSG00000057969   150.693        2.61724  0.285023   9.18255 4.20997e-20
##                         padj
##                    <numeric>
## ENSMUSG00000029843 9.80312e-38
## ENSMUSG00000095562 1.92437e-28
## ENSMUSG00000039004 1.74756e-27
## ENSMUSG00000004105 5.09782e-18
## ENSMUSG00000022150 5.09782e-18
## ENSMUSG00000057969 1.27611e-16
```

```
plot(res_1$log2FoldChange,-log10(res_1$padj),pch=20,
     xlim = c(-5,10), ylim=c(0,15),
     xlab = "log2FoldChange",
     ylab = "-log10(FDR)")

#with(subset(res_1, padj<0.05 & abs(log2FoldChange)>2), points(log2FoldChange, -log10(padj), pch=20, col="red", c
ex=0.5))
with(subset(res_1, padj<0.05), points(log2FoldChange, -log10(padj), pch=20, col="red", cex=0.5))

#Add lines for absolute FC>2 and FDR Q<0.05
abline(v=0, col="black", lty=3, lwd=1.0)
abline(v=-2, col="black", lty=4, lwd=2.0)
abline(v=2, col="black", lty=4, lwd=2.0)
abline(h=-log10(0.05), col="black", lty=4, lwd=2.0)
```

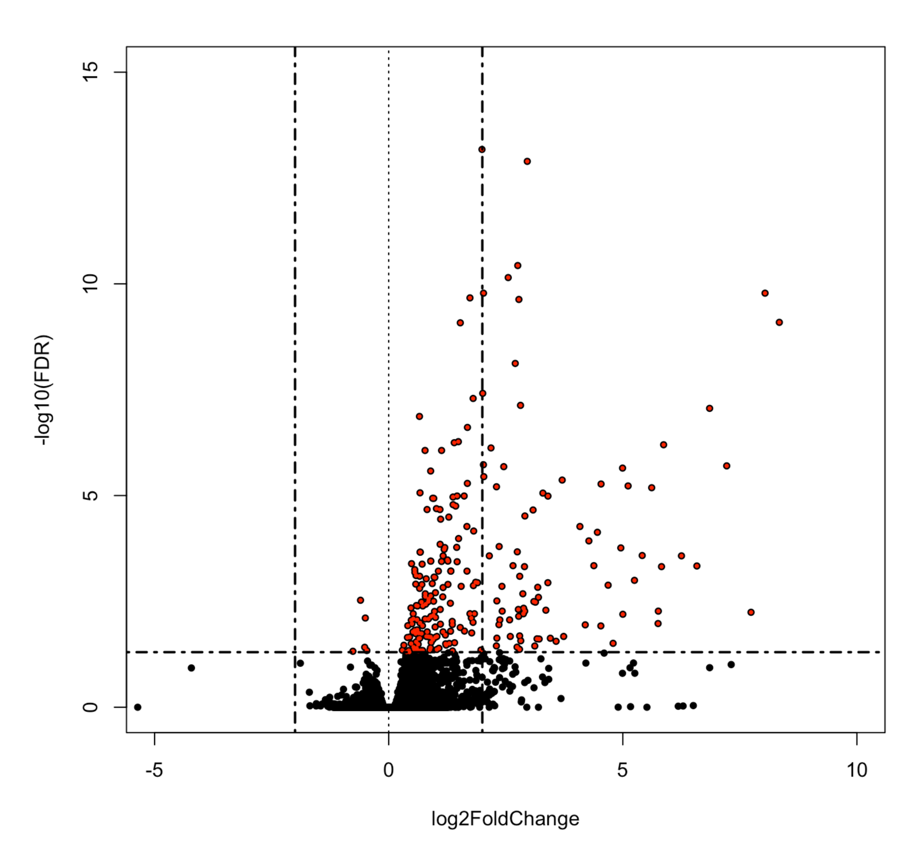

Detect DE genes between WT_Vehicle and WT_Nutlin3

```
subset2_idx=(as.character(type)=="wildtype")
dds_2 <- DESeqDataSetFromMatrix(countData = counts[,subset2_idx],
                              colData = coldata[subset2_idx,],
                              design = ~ condition)
dds_2 <- DESeq(dds_2)
res_2 <- results(dds_2)
summary(res_2) #up: 0%, down: 0%
```

```
##
## out of 22585 with nonzero total read count
## adjusted p-value < 0.1
## LFC > 0 (up)       : 0, 0%
## LFC < 0 (down)     : 0, 0%
## outliers [1]       : 43, 0.19%
## low counts [2]     : 0, 0%
## (mean count < 0)
## [1] see 'cooksCutoff' argument of ?results
## [2] see 'independentFiltering' argument of ?results
```

```
# Result is stored in "resOrdered2"
resOrdered2=res_2[order(res_2$padj),]
#write.csv(as.data.frame(resOrdered2), file="Veh_vs_Nut(WT).csv") # this line is to write result into .csv file
#resLFC_2 <- lfcShrink(dds_2, coef="condition_Nutlin_3_vs_Vehicle", type="apeglm")
#resLFC_2

# check the first 5 lines of result
head(resOrdered2)
```

```
## log2 fold change (MLE): condition Nutlin 3 vs Vehicle
## Wald test p-value: condition Nutlin 3 vs Vehicle
## DataFrame with 6 rows and 6 columns
##                     baseMean log2FoldChange     lfcSE      stat      pvalue
##                    <numeric>      <numeric> <numeric> <numeric>   <numeric>
## ENSMUSG00000041323   93.326905       0.927672 0.2420101  3.833195 1.26489e-04
## ENSMUSG00000027562 3187.081430       0.264813 0.0679588  3.896669 9.75246e-05
## ENSMUSG00000032554 4782.427285       0.344786 0.0896721  3.844964 1.20570e-04
## ENSMUSG00000049353   13.769472       2.849383 0.7598489  3.749934 1.76881e-04
## ENSMUSG00000016498    1.819579       3.176697 1.9762573  1.607431 1.07960e-01
## ENSMUSG00000028825    0.890638       1.929234 2.6662274  0.723582 4.69323e-01
##                         padj
##                    <numeric>
## ENSMUSG00000041323  0.950441
## ENSMUSG00000027562  0.950441
## ENSMUSG00000032554  0.950441
## ENSMUSG00000049353  0.996815
## ENSMUSG00000016498  0.999899
## ENSMUSG00000028825  0.999899
```

```
plot(res_2$log2FoldChange,-log10(res_2$padj),pch=20,
     xlab = "log2FoldChange",
     ylab = "-log10(FDR)",
     xlim = c(-10,10))

#with(subset(res_2, padj<0.05 & abs(log2FoldChange)>2), points(log2FoldChange, -log10(padj), pch=20, col="red", c
ex=0.5))
with(subset(res_2, padj<0.05), points(log2FoldChange, -log10(padj), pch=20, col="red", cex=0.5))

#Add lines for absolute FC>2 and FDR Q<0.05
abline(v=0, col="black", lty=3, lwd=1.0)
abline(v=-2, col="black", lty=4, lwd=2.0)
abline(v=2, col="black", lty=4, lwd=2.0)
abline(h=-log10(0.05), col="black", lty=4, lwd=2.0)
```

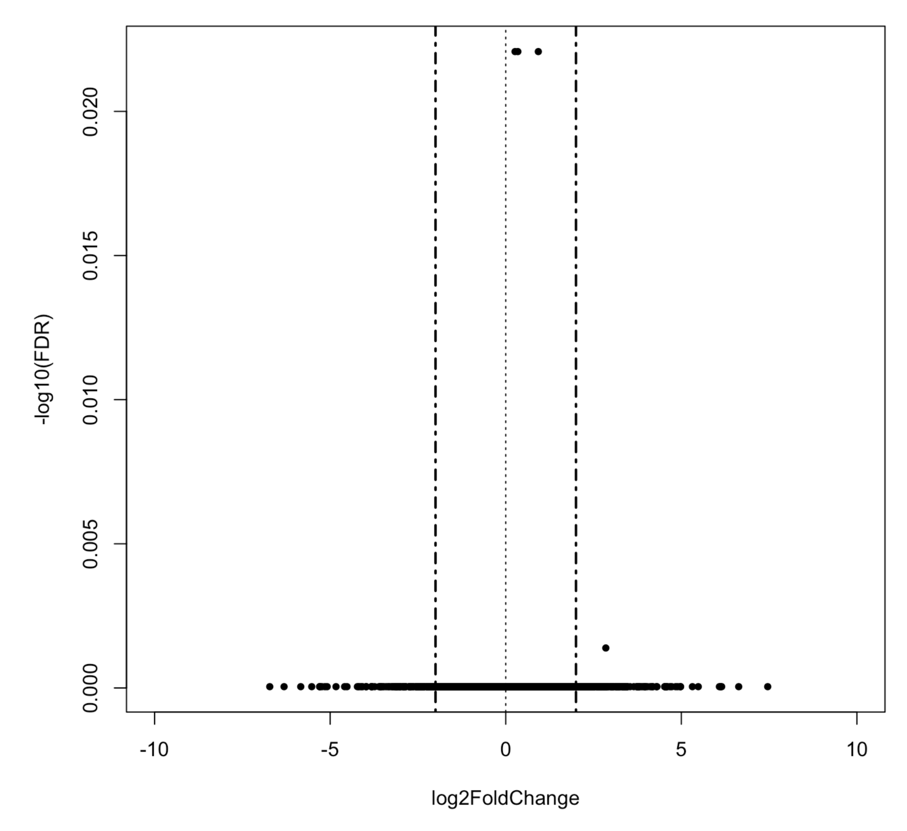

Detect DE genes between WT_Vehicle and KO_Vehicle

```
subset3_idx=(as.character(condition)=="Vehicle")
dds_3 <- DESeqDataSetFromMatrix(countData = counts[,subset3_idx],
                              colData = coldata[subset3_idx,],
                              design = ~ type)
dds_3 <- DESeq(dds_3)
res_3 <- results(dds_3)
summary(res_3) #up: 0.049%, down: 0.089%
```

```
##
## out of 22578 with nonzero total read count
## adjusted p-value < 0.1
## LFC > 0 (up)       : 11, 0.049%
## LFC < 0 (down)     : 20, 0.089%
## outliers [1]       : 16, 0.071%
## low counts [2]     : 0, 0%
## (mean count < 0)
## [1] see 'cooksCutoff' argument of ?results
## [2] see 'independentFiltering' argument of ?results
```

```
# Result is stored in "resOrdered3"
resOrdered3=res_3[order(res_3$padj),]
#write.csv(as.data.frame(resOrdered3), file="WT_vs_KO(Veh).csv") # this line is to write result into .csv file
#resLFC_3 <- lfcShrink(dds_3, coef="type_knock_out_vs_wildtype", type="apeglm")
#resLFC_3

# check the first 5 lines of result
head(resOrdered3)
```

```
## log2 fold change (MLE): type knock out vs wildtype
## Wald test p-value: type knock out vs wildtype
## DataFrame with 6 rows and 6 columns
##                     baseMean log2FoldChange     lfcSE      stat      pvalue
##                    <numeric>      <numeric> <numeric> <numeric>   <numeric>
## ENSMUSG00000000838  1017.144      -1.179192 0.0860293 -13.70687 9.23581e-43
## ENSMUSG00000095562   109.370       7.039586 0.6749312  10.43008 1.80737e-25
## ENSMUSG00000026837   433.304      -0.995107 0.1366851  -7.28029 3.33109e-13
## ENSMUSG00000039474  2023.626      -0.437512 0.0739895  -5.91316 3.35596e-09
## ENSMUSG00000024793   880.178       0.675892 0.1192620   5.66729 1.45073e-08
## ENSMUSG00000041444  3351.765      -0.307535 0.0577925  -5.32137 1.02987e-07
##                         padj
##                    <numeric>
## ENSMUSG00000000838 2.08378e-38
## ENSMUSG00000095562 2.03890e-21
## ENSMUSG00000026837 2.50520e-09
## ENSMUSG00000039474 1.89293e-05
## ENSMUSG00000024793 6.54627e-05
## ENSMUSG00000041444 3.87265e-04
```

```
plot(res_3$log2FoldChange,-log10(res_3$padj),pch=20, xlim=c(-8,8),
     xlab = "log2FoldChange",
     ylab = "-log10(FDR)")

#with(subset(res_3, padj<0.05 & abs(log2FoldChange)>2), points(log2FoldChange, -log10(padj), pch=20, col="red", c
ex=0.5))

with(subset(res_3, padj<0.05), points(log2FoldChange, -log10(padj), pch=20, col="red", cex=0.5))

#Add lines for absolute FC>2 and FDR Q<0.05
abline(v=0, col="black", lty=3, lwd=1.0)
abline(v=-2, col="black", lty=4, lwd=2.0)
abline(v=2, col="black", lty=4, lwd=2.0)
abline(h=-log10(0.05), col="black", lty=4, lwd=2.0)
```

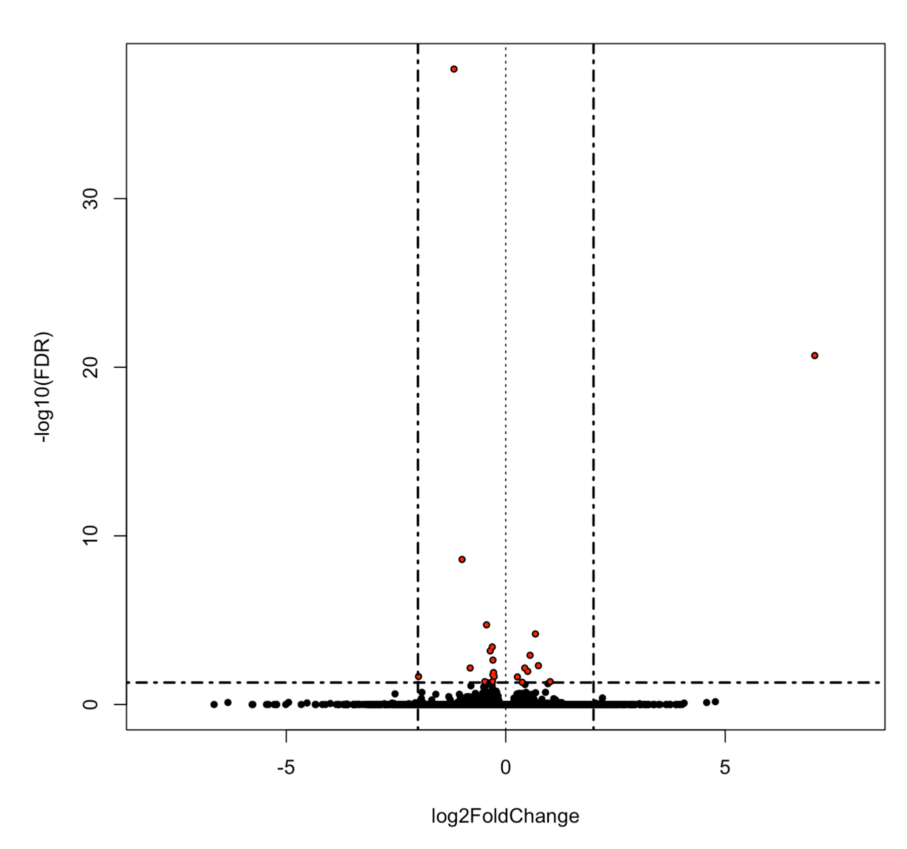

Detect DE genes between WT_Vehicle and KO_Nutlin

```
subset4_idx=c(1:3,10:12)
con = c(rep("WV",3),rep("KN",3))
con = factor(con,levels = c("KN","WV"))
dds_4 <- DESeqDataSetFromMatrix(countData = counts[,subset4_idx],
                              colData = data.frame(con=con),
                              design = ~con)
dds_4 <- DESeq(dds_4)
res_4 <- results(dds_4)
summary(res_4) #up: 0.19%, down: 1.2%
```

```
##
## out of 22591 with nonzero total read count
## adjusted p-value < 0.1
## LFC > 0 (up)       : 42, 0.19%
## LFC < 0 (down)     : 282, 1.2%
## outliers [1]       : 28, 0.12%
## low counts [2]     : 2627, 12%
## (mean count < 4)
## [1] see 'cooksCutoff' argument of ?results
## [2] see 'independentFiltering' argument of ?results
```

```
# Result is stored in "resOrdered4"
resOrdered4=res_4[order(res_4$padj),]
#write.csv(as.data.frame(resOrdered4), file="KOnut_vs_WTveh.csv") # this line is to write result into .csv file
#resLFC_4 <- lfcShrink(dds_4, coef="con_WV_vs_KN", type="apeglm")
#resLFC_4

# check the first 5 lines of result
head(resOrdered4)
```

```
## log2 fold change (MLE): con WV vs KN
## Wald test p-value: con WV vs KN
## DataFrame with 6 rows and 6 columns
##                     baseMean log2FoldChange     lfcSE      stat      pvalue
##                    <numeric>      <numeric> <numeric> <numeric>   <numeric>
## ENSMUSG00000000838   985.054       1.298570  0.110185  11.78537 4.64357e-32
## ENSMUSG00000068323   214.185      -8.022474  0.803928  -9.97910 1.88169e-23
## ENSMUSG00000061808 23818.651      -8.545720  0.961921  -8.88401 6.44909e-19
## ENSMUSG00000048108   376.376      -7.719960  1.133667  -6.80972 9.77870e-12
## ENSMUSG00000027962   502.579      -0.854853  0.127393  -6.71037 1.94132e-11
## ENSMUSG00000025350   102.957      -2.643986  0.395930  -6.67791 2.42372e-11
##                         padj
##                    <numeric>
## ENSMUSG00000000838 9.25741e-28
## ENSMUSG00000068323 1.87567e-19
## ENSMUSG00000061808 4.28564e-15
## ENSMUSG00000048108 4.87370e-08
## ENSMUSG00000027962 7.74043e-08
## ENSMUSG00000025350 8.05323e-08
```

```
plot(res_4$log2FoldChange,-log10(res_4$padj),pch=20, xlim=c(-12,10),
     xlab = "log2FoldChange",
     ylab = "-log10(FDR)")

#with(subset(res_4, padj<0.05 & abs(log2FoldChange)>2), points(log2FoldChange, -log10(padj), pch=20, col="red", c
ex=0.5))
with(subset(res_4, padj<0.05 ), points(log2FoldChange, -log10(padj), pch=20, col="red", cex=0.5))

#Add lines for absolute FC>2 and FDR Q<0.05
abline(v=0, col="black", lty=3, lwd=1.0)
abline(v=-2, col="black", lty=4, lwd=2.0)
abline(v=2, col="black", lty=4, lwd=2.0)
abline(h=-log10(0.05), col="black", lty=4, lwd=2.0)
```

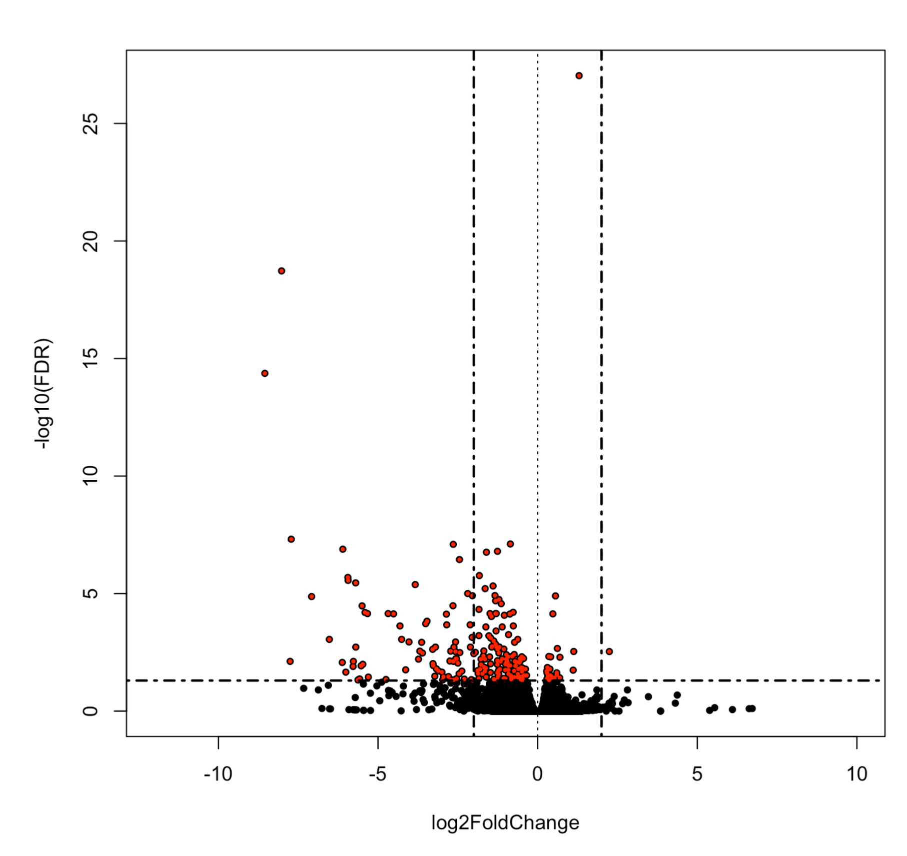

Supplement: Supplementary file 3 — Additional file 3: Code S3. R Codes for volcano plot. [file 12916_2022_2370_MOESM3_ESM.pdf]
