# Supplementary material for: Sustained correction of hippocampal neurogenic and cognitive deficits after a brief treatment by Nutlin-3 in a mouse model of fragile X syndrome
Source: BMC Med. 2022 May 13;20:163. doi: 10.1186/s12916-022-02370-9 (PMC9103116; doi:10.1186/s12916-022-02370-9)
Supplement: Supplementary file 9 — Additional file 9: Fig. S1. Transient treatment with Nutlin-3 has long-lasting rescue effect on cognitive deficits in mice with selective deletion of Fmr1 in adult new neurons. Fig. S2. Acetylated Histone H3 levels in the hippocampus and the cortex. Fig. S3. Boxplot to show the density distribution of raw log-intensities of RNA-seq data of all samples. Fig. S4. Volcano plots showing gene fold changes and adjusted P-values between different conditions. Fig. S5. GO analysis of top upstream TF. [file 12916_2022_2370_MOESM9_ESM.pdf]

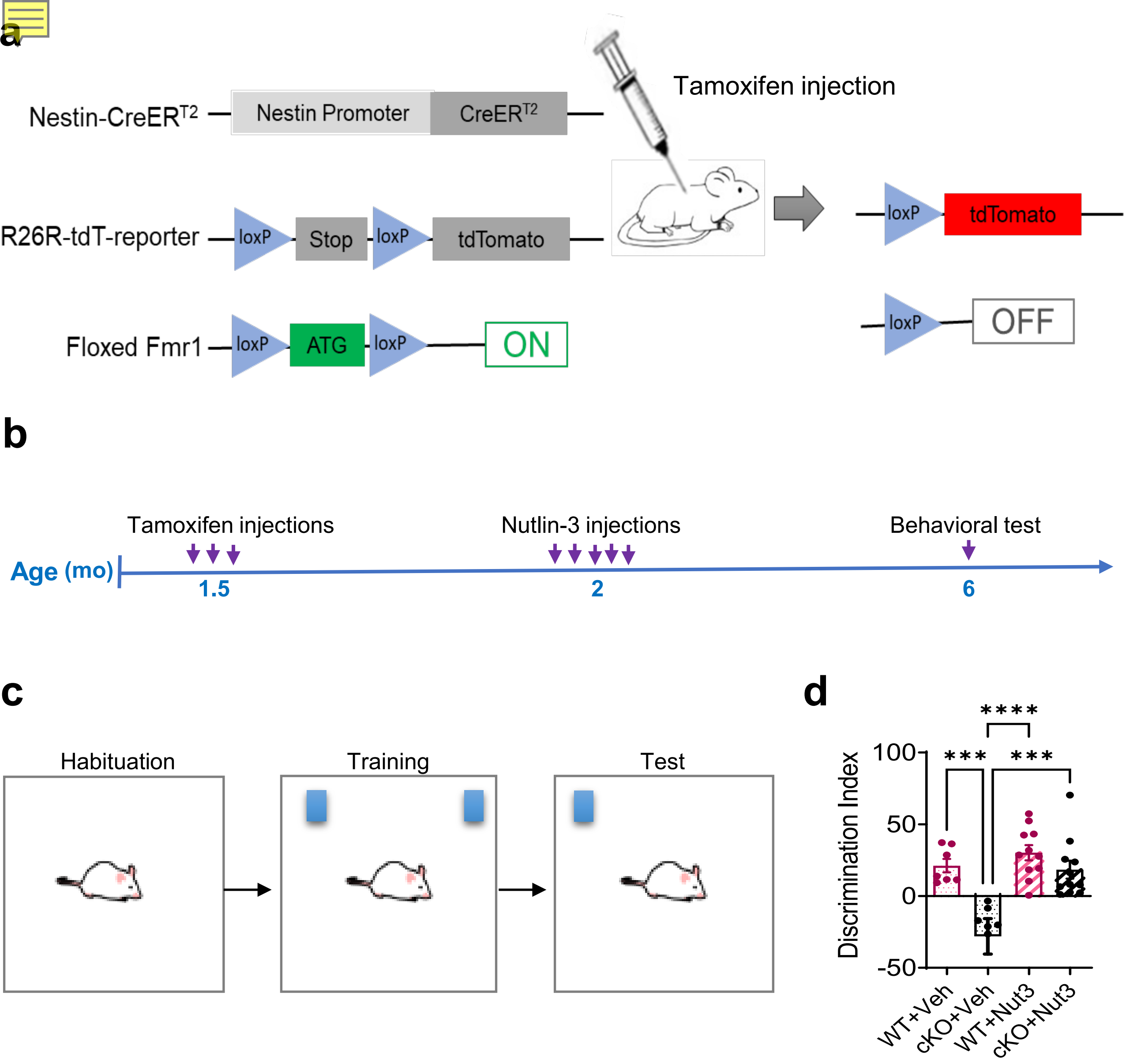

FigureS1: Transient treatment with Nutlin-3 has long-lasting rescue effect on cognitive deficits in mice with selective deletion of Fmr1 in adult new neurons. a An inducible FMRP conditional knockout mouse line was created by crossing Nestin-CreER^{T2} mice, ROSA26-STOP-tdTomato (Ai14) mice and *Fmr1* floxed (*Fmr1* cKO) mice. Administration of tamoxifen to adult mice results in the removal of the first exon of the mouse *Fmr1* gene and the "Stop" codon before tdTomato (tdT) in Nestin-expressing cells and their subsequent progenies. **b** Experimental scheme for analyzing cognitive performances in *Fmr* cKO treated with Nutlin-3 or vehicle **c** Schematic of novel location recognition test for assessing spatial memory in *Fmr1* cKO mice. **d** Nutlin-3 treatment fully rescued spatial memory deficits in *Fmr1* cKO mice 4 months after injection (n = 6 to 11 mice per group). ***P < 0.001. Data are presented as means ± SEM.

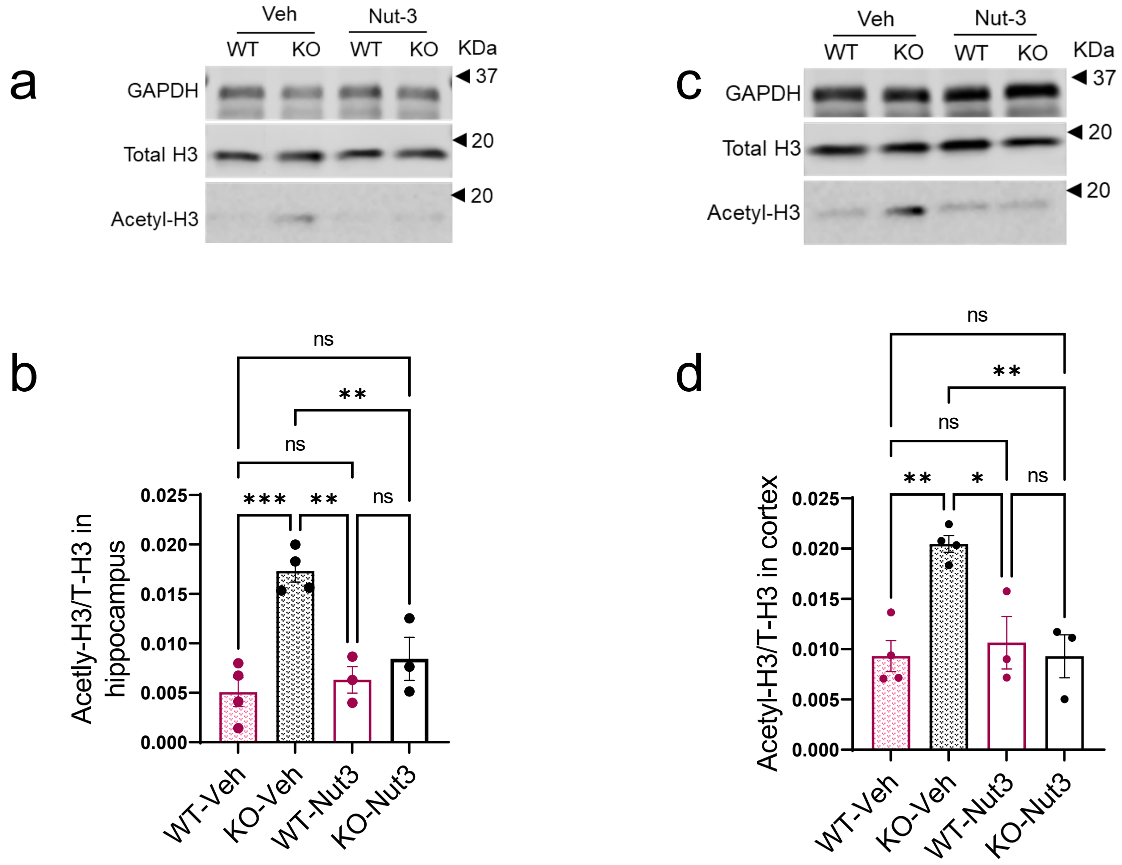

Figure S2. **Acetylated H3 levels in the hippocampus and the cortex**
Western blot analysis of Acetylated H3 (Acetyl-H3) levels in the hippocampal (a,b) and cortex (c,d) tissues of *Fmr1* KO and WT 4-months after Nutlin-3 or vehicle treatment (n = 4). Total H3 (T-H3) was used as loading control. *P < 0.05; **P < 0.01 ***P < 0.001. Data are presented as means ± SEM.

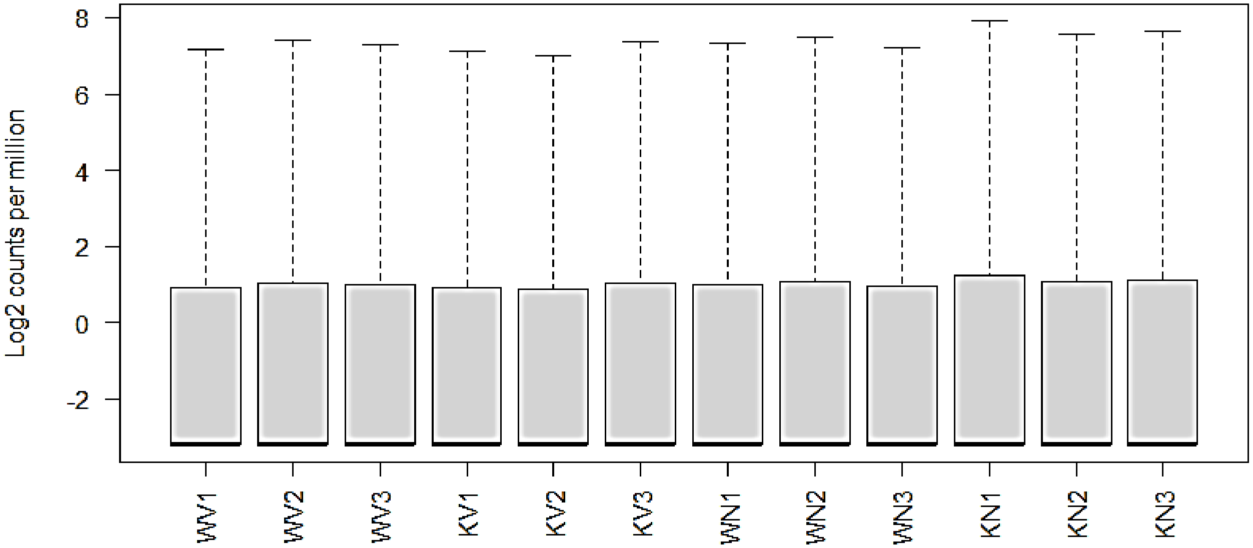

Figure S3: Boxplot to show the density distribution of raw log-intensities of RNA-seq data of all samples.

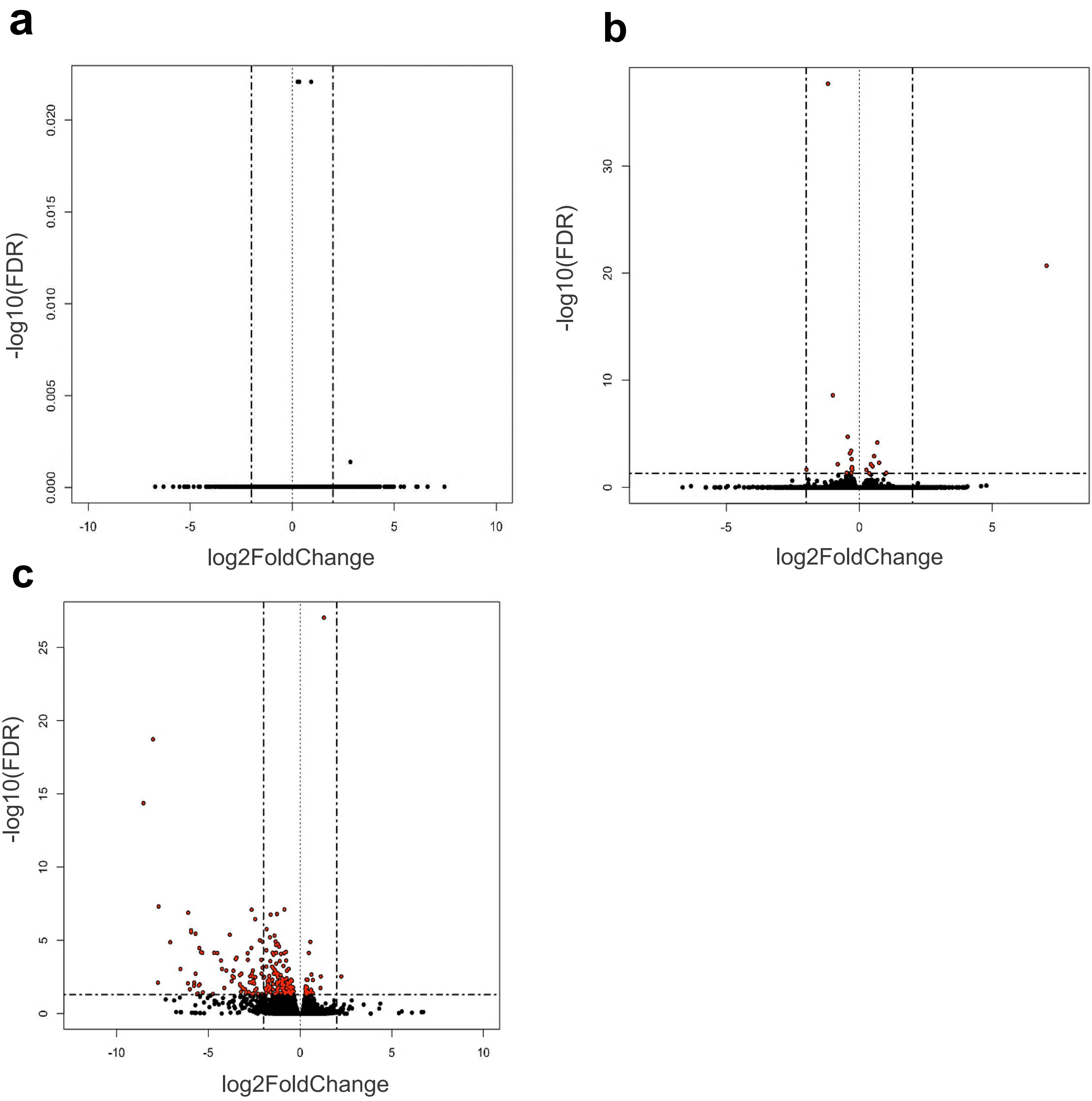

Figure S4. Volcano plots showing gene fold changes and adjusted P-values between different conditions.
Comparisons were made between WT-Vehicle and WT-Nutlin3 (a), WT-Vehicle and KO-Vehicle (b), and between WT-Vehicle and KO-Nutlin-3 (c) . The most upregulated genes are towards the right, the most downregulated genes are towards the left, and the most statistically significant genes are towards the top. The red points are the genes with adjusted P-value < 0.05.

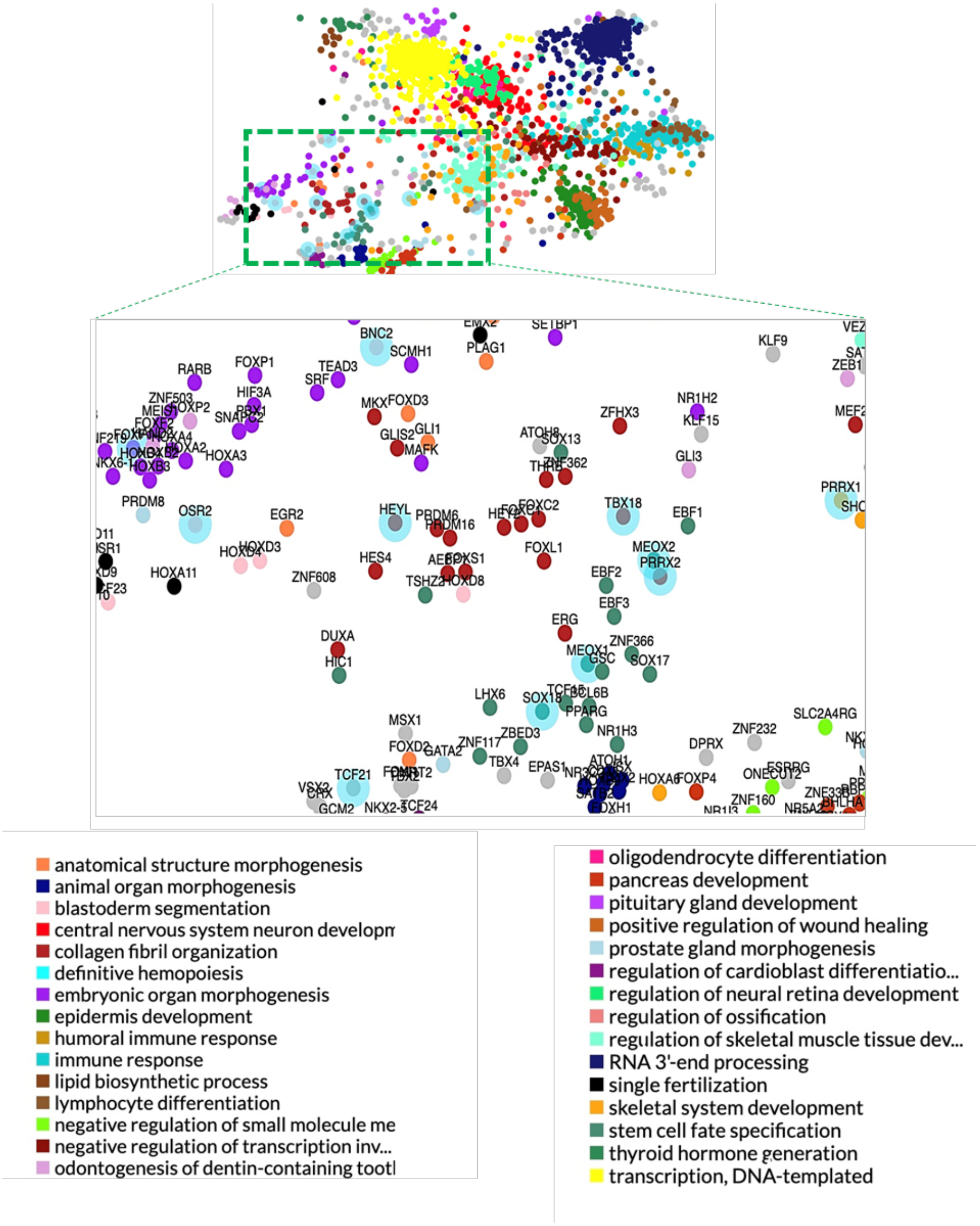

Figure S5. **GO analysis of top upstream TF.**
ChEA3-generated GO enrichment analysis of top upstream TFs ranked by ChEA3, using average integrated ranks across all libraries, for DEGs between Fmr1 KO treated with Nutlin-3 vs vehicle.
